# Smartphone Application in Postgraduate Clinical Psychology Training: Trainees’ Perspectives

**DOI:** 10.3390/ijerph16214206

**Published:** 2019-10-30

**Authors:** Carol C. Choo, Bhavani Devakaran, Peter K. H. Chew, Melvyn W. B. Zhang

**Affiliations:** 1 Department of Psychology, College of Healthcare Sciences, James Cook University, Singapore 387380, Singapore; ccychoo@gmail.com (C.C.C.); bhavani.devakaran@my.jcu.edu.au (B.D.); peter.chew@jcu.edu.au (P.K.H.C.); 2 College of Healthcare Sciences, Division of Tropical Health and Medicine, James Cook University, Queensland 4811, Australia; 3 Family Medicine and Primary Care, Lee Kong Chian School of Medicine, Nanyang Technological University, Singapore 308232, Singapore

**Keywords:** clinical psychology education, self-efficacy, smartphone application, mixed methods, m-learning

## Abstract

M-learning refers to the learning that takes advantage of mobile technologies. Although research shows enhanced educational outcomes from m-learning in some Asian countries, the generalizability to postgraduate clinical psychology training in Singapore remains unclear. Current professional standards in clinical psychology training emphasize the importance of attainment of clinical competencies in trainees. Although learning theories indicated potential for m-learning to be incorporated into the local clinical psychology curriculum, trainees’ perspectives have not been adequately explored on m-learning. The study aimed to address this gap by exploring the use of m-learning via a novel smartphone application in clinical psychology training using mixed-methods design. Eight clinical psychology trainees between the ages of 26 to 43 years old (mean age of 31.75, SD = 5.49) enrolled in a relevant coursework subject were recruited. Participants were randomly allocated to the experimental and control groups. The experimental group accessed the novel application weekly, from week 1 to week 6, and participants in the control group accessed the application after week 6. Participants from both groups completed a brief demographic questionnaire, and the following scales New General Self-Efficacy Scale adapted for Education (NGSES-E) and self-reported scale of learning outcomes (SLO). The qualitative study explored how participants perceived and experienced the novel application. Participants from the experimental group were invited to provide open-ended responses about the novel application. Data were analyzed using thematic analysis. Results from the qualitative analysis yielded four themes of: Convenience, preferred learning style, building confidence, and putting theory into practice. Findings from the qualitative study were consistent with previous studies about advantages of m-learning: That the e-platform was convenient, the learning style was engaging, which helped to build confidence, and facilitate practical learning of skills. The qualitative results were helpful in understanding the users’ perspectives and experience of the novel application, indicating that future research in this innovative area is necessary. However, the quantitative outcomes were not significant, limitations would be discussed, and recommendations made for future research.

## 1. Introduction

With the rising trend in e-technology [1,2], the advancement of smartphones has ushered in an era where smartphone applications can be used to enhance teaching and learning [3,4]. M-learning is often referred to as the learning that takes advantage of mobile technologies, to occur without being limited to a fixed place [5]. M-learning facilitates students’ continuous learning beyond the physical constraints of the traditional classroom [6]. Previous research showed that m-learning could incorporate the benefits of smartphones, such as portability, accessibility, and interactive features [4,7]. Studies have reported how the affordability and ease of using smartphones as compared to other mobile technologies have maximized students’ opportunities to gain knowledge inside and outside of classrooms [8]. Learning and educational outcomes could be enhanced when the modalities are engaging [9] and appeal to the learner.

In addition, Ayres et al. [10] suggested that learning new skills via mobile technology yielded positive outcomes that could feed into components of self-confidence and self-determination. One such component included self-efficacy (SE). SE in education refers to an individual’s belief in his/her capability to perform or achieve certain academic tasks or goals [11,12,13]. According to Bandura’s [14] social cognitive theory of SE, personal mastery, vicarious learning, physiological states and verbal persuasion are the four determinants of SE [13]. Research indicated that personal mastery, such as experiencing achievements, and vicarious learning, such as watching someone else’s competent performance [15], are the most powerful information sources of SE.

SE beliefs are sensitive to vicarious learning, particularly when the observing students have little experience (especially mastery experience) with a skill, they become reliant on vicarious experiences to learn a required skill [15]. Huang [12] also reported that when lacking experience or opportunities for self-mastery, vicarious learning served as the most significant determinant of SE. Educational videos available online could be one example of the integration of m-learning and vicarious learning to enhance contemporary education. These will be described below.

According to the Cognitive Theory of Multimedia Learning, an active learning process is more successful via the dual channel model [16]. Consistent with the Cognitive Theory of Multimedia learning, Buzzetto-More [17] postulated that educational videos were effective in constructing knowledge and building memory. These digital videos, incorporated as part of a learning tool, have been shown to increase levels of student engagement and experiential learning, through visual, audio, and kinesthetic modes [18]. There is potential for educators to harness the benefits of video resources, such as instructional videos for use as an adjunct tool in education [17]. Such video resources delivered via m-learning modality could be based on activities incorporating combinations of learning from both digital-world and real-world learning resources [5]. Such activities could involve roleplaying, where key skills are demonstrated, which could also tap upon the principles of Bandura’s [14] learning theories, and vicarious learning, to enhance students’ SE, particularly in clinical skills training. SE in students could be enhanced when the students observe their peers’ competent performance [14,19]. Educational videos incorporating a peer’s competent demonstration of clinical skills might address the hands-on practical components that a traditional classroom setting may not be able to reproduce. In addition, it was postulated that the greater the degree of similarity to the model as perceived by the learner, the stronger would be the influence of observational learning on academic SE. Observing a competent peer performing required competencies and skills had been shown to enhance SE in education, which would, in turn, lead to enhanced performance in academic competencies and skills [15]. This association can be explained by the influence of academic SE on a student’s motivation, efforts, use of learning strategies, accomplishments, and performance [17]. Academic SE, in turn, influences a student’s academic satisfaction and transforms their school and career-related aspirations [20].

The literature reviewed showed evidence for enhanced educational outcomes from m-learning in some Asian countries, such as Thailand [21]. However, the studies reviewed lacked a specific definition of educational outcomes [22] that could be applied for postgraduate clinical psychology education in Singapore. None of the studies reviewed on m-learning was done for postgraduate clinical psychology trainees in Singapore; hence, the results may not be generalizable to the local postgraduate clinical psychology curriculum. In view of the current professional standards in clinical psychology training, which emphasize the importance of the attainment of clinical competencies within the clinical internship, whereby clinical competencies and skills need to be acquired and demonstrated, e.g., when conducting therapy with clients [23], there is potential for m-learning to be incorporated into the local clinical psychology curriculum.

Majority of the studies reviewed used quantitative designs that explored the outcome variables by assessing students’ attitudes [24], level of engagement [9] and academic performance [25]. Qualitative studies would be valuable to explore learners’ perspectives on the use of m-learning in clinical psychology education in the local context. Educational strategies that take into account learners’ perspectives and learning preferences would enhance their effectiveness and contribute to better educational outcomes [26]. The findings of this study could, hence, shed light on future innovations, to inform and enhance teaching and training efforts in postgraduate programs in clinical psychology in Singapore using m-learning delivered via a smartphone application.

Exploring learners’ perspectives could inform the development of such e-resources [27,28], before such e-resources are integrated into an academic curriculum [4]. However, there are limited local studies exploring m-learning delivered via smartphone applications in education that catered to students’ needs, expectations and learning preferences. Moreover, the literature search also indicated that there is a lack of studies exploring the use of smartphone application in postgraduate clinical psychology training in Singapore.

This study aimed to address this gap in the local literature by exploring clinical psychology trainees’ perspectives on a novel smartphone application in clinical psychology training through a mixed-methods study design. As recommended by previous research [4], the employment of mixed-methods study design will be useful to explore the impact of m-learning utilizing the novel smartphone application. A mixed-methods approach in this study entails the use of both quantitative and qualitative methods. To strengthen the qualitative paradigm, we will explore the triangulation of the qualitative findings with the quantitative outcomes, with the overall objective to explore, describe and explain meanings, perspectives and experience of trainees on the novel smartphone application. The overall outcomes will be reported in this formative study on the use of the novel application, from a 6-week pilot study among local clinical psychology trainees. Since the novel application is being evaluated for the first time in the local clinical psychology education context, relatively small sample size is utilized to explore the acceptability of the approach before an expensive randomized controlled trial is considered. It is anticipated that findings from this preliminary study will contribute to early evidence of acceptance and use of m-learning, and its association with the intended educational outcomes, as well as troubleshooting, if any, to inform future improvements.

### 1.1. Qualitative Study

The qualitative study was exploratory and formative in nature, with the aim to explore the perceptions and experience of participants who had used the novel application. The research question was “How do participants perceive and experience the novel smartphone application?”

### 1.2. Quantitative Study

It was envisaged that qualitative outcomes would be triangulated with the quantitative findings on trainees’ perspectives and experience of the novel smartphone application. The research question was “what is the effect of the novel smartphone application on clinical psychology trainees’ educational outcomes, specifically levels of academic self-efficacy (SE) and self-reported learning outcomes (SLO) in education?” It was hypothesized that participants who did and did not use the novel smartphone application would differ significantly in academic SE and SLO levels, specifically, participants in the experimental group who used the novel smartphone application would have increased academic SE and SLO levels as compared to participants in control group (who did not use the novel smartphone application).

## 2. Methods

### 2.1. Participants

The participants were students enrolled in a clinical coursework subject in a postgraduate psychology program at a medium-sized university in Singapore. These students were randomly allocated into two groups (4 in the control group and 4 in the experimental group). Eight participants (87.5% females and 12.5% males) completed the study and their ages ranged from 26 to 43 years (M = 31.75, SD = 5.49).

### 2.2. Measures

The self-reported scale of learning outcomes (SLO) was developed to address the research question of “what is the effect of the novel smartphone application on clinical psychology trainees’ educational outcomes, specifically Self-reported Learning Outcomes (SLO) in education”. Psychometric properties are not currently available.

The SLO comprises three items whereby participants rate how much of the following outcomes have been achieved on a scale of 1–100%. The SLO is scored by summing the total ratings for items 1 to 3. The potential range of scores is between 0 to 300%, with higher total scores indicating higher SLO. The items are:

1. I have acquired knowledge about the application of assessment skills.

2. I have acquired knowledge about the application of therapy skills.

3. I have acquired knowledge about the application and integration of micro skills into working with clients.

In addition to its brevity, the New General Self-Efficacy Scale (NGSES) has good psychometric properties, as compared with other measures of SE [29,30]. The NGSES demonstrated good internal consistency (ranging from 0.85 to 0.90), that are above the generally accepted 0.70 cutoff for exploratory research. It had good test-retest reliability in three time intervals within 67 days, ranging between *r* = 0.62 to *r* = 0.65 [29,31,32].

Academic SE assesses domain-specific beliefs [12]. In the current study, the NGSE has been adapted for education purposes (NGSES-E). It comprises eight items that assess how much participants believe they can achieve their academic goals, despite difficulties. An example item is, “I will be able to achieve most of the academic goals that I have set for myself”. The NGSES-E uses 5-point Likert scale ranging from (1) strongly disagree to (5) strongly agree. Higher scores indicate higher levels of SE.

### 2.3. Procedure

Ethics approval was obtained from the human research ethics committee at the university which hosted the study (Human Ethics Approval Number: H6934). Purposeful sampling was used. Students enrolled in a relevant clinical postgraduate coursework subject were recruited via email announcements with information about the study. Prior to the start of the study, participants were provided with an information sheet and an informed consent form. Participants were notified that their participation was voluntary, and they had the right to withdraw at any time without explanation or prejudice. After participants provided consent, participants were then contacted by the researcher.

### 2.4. Material

An experienced clinical psychology educator and clinical psychologist (primary author) developed the novel smartphone application for postgraduate clinical psychology training in Singapore, in collaboration with an industry partner. To the best of our knowledge, this is the only smartphone application that has been developed locally for postgraduate clinical psychology education. The application was developed to be in addition to the usual lecture materials and face-to-face lectures. The application was designed to be an adjunct tool in addition to the coursework subjects that students undertook in the postgraduate program. As the program included clinical internship involving a clinical placement at the psychology clinic on campus, the roleplay sessions were filmed on location, at the clinic. Both research assistants were graduates from the local psychology postgraduate program and were trained by the primary author. The videos featured research assistants in the roles of the psychologist and the ‘pseudo’-patient (with a mental health problem).

Six video clips were embedded in this application. The video clips depicted six mock sessions whereby clinical assessment and brief therapy was conducted, which followed the internal protocols at the psychology clinic. Six sessions were deemed suitable for a brief intervention, to address the mental health problem in the case. The content of the case study used was based on a de-identified local case developed for educational purposes. An external collaborator helped to embed the training videos into the novel smartphone application and provided access on e-platform. A low-cost method utilizing web based technologies was used in the development of the application via the online platform. The content of the application was intended to be in addition to the available subject materials.

#### 2.4.1. Quantitative Process

This part of the study involved a quantitative process whereby eight participants were randomly assigned to two groups: Experimental group (group 1) and control group (group 2). Participants in the experimental group were asked to access the novel smartphone application in addition to the usual subject materials in week 1. Participants in the control group were asked to access the smartphone application after week 6, after data collection was completed.

In week 1, participants in the experimental group (group 1) were informed about their allocation to group 1, and they were asked to not share the smartphone application with others during the experimental period of six weeks. Participants were given the Uniformed Resource Locator (URL) to access the smartphone application along with week 1 password through email. Participants were informed to access the URL each week with a different password, provided weekly throughout the six weeks. Participants in the control group (group 2) were informed about their allocation to group 2 and that they would receive the link to the smartphone application after week 6. They were given access to the smartphone application after week 6, after data collection was completed, and then they were thanked for their participation.

In week 1, participants in both groups (groups 1 and 2) were asked to complete a brief demographics questionnaire, the NGSES-E and SLO. All participants in both groups were reminded to complete the two scales, NGSES-E and SLO in weeks 3 and 6 as well.

#### 2.4.2. Qualitative Process

In week 6, only the participants in the experimental group (who had used the application) were asked to provide open-ended written responses about their views of the novel smartphone application. All participants from the experimental group gave their written responses.

Six phases of analysis were employed [33]: Familiarizing with the data, generating initial codes, searching for themes, reviewing themes, defining and naming themes, and final reporting using selected extracts. The textual data provided by the participants was re-read several times by the researchers, resulting in data immersion, before progressing into the coding phase. The coding involved an inductively driven process, whereby the textual data was read and re-read. Codes identified the most basic features of the raw data, such as keywords and phrases that appeared relevant and pertinent to the research question. The coding process involved a constant moving backwards and forwards, within the textual data set, in order to analyze the extracts that had been initially identified. Rigorous note-taking was undertaken in the coding process, and coding schemes were identified through the annotation of ideas. The codes were then sorted according to their similarity using a thematic map, into identified themes, which would serve as the units of analysis. Further reviews were made to ensure no codes had been omitted. All initial codes relevant to the research question were incorporated into a theme.

A theme captures something important and meaningful within the data set in relation to the research question [33]. Based on the context of this study, a theme would have to relate to this research topic on exploring participant’s perception and experience of using the novel application. After a set of candidate themes had been established, the refinement of the themes was necessary. Consistent with expert guidelines [33], this process involved reviewing the collated codes and extracts, looking for internal homogeneity, and considering whether the set of candidate themes formed a coherent pattern and accurately reflected what was evident in the data set as a whole.

Additionally, identifiable associations, links, and distinctions between themes were detected. The practical basis for this thematic refinement process was to ensure that the themes were all broadly related to one another, yet did not overlap too closely in their content. Conceptually, this meant that the themes were able to stand in their own discernable categories with regards to the original patterns that had emerged in each. The following stage involved defining and naming the themes, with clear definition accompanied by a detailed analysis. The final stage involved choosing examples to illustrate elements of the themes. The extracts will be included in the results section. These extracts were selected as they clearly identified issues within the theme and presented a comprehensible example of the point being made.

## 3. Results

### 3.1. Qualitative Analysis

Similar to Fernandes et al. [34], two researchers coded the scripts independently and manually using a thematic matrix technique, within the framework of inductive coding. On completion of the coding, analyses were compared, any discrepancies were considered, and a consensus was reached.

The thematic analysis process that was applied to the textual data elicited key concepts that were evident in the data. The codes were categorized into the following four themes. The subsections below include the extracts, which captured the essence of the respective theme without unnecessary complexity [33]:

1. Convenience;
2. Preferred learning style;
3. Building confidence;
4. Putting theory into practice.

#### 3.1.1. Convenience

This theme captured participants’ perspectives of ease and effortlessness, which illustrated the benefits of the e-modality and flexible medium for accessing the learning content.

P1: “Smartphone application is very helpful”, “enhancing our learning”;

P3: “Great flexibility for students to access necessary content”, “quick”, and “user friendly”;

P4: “Convenient platform to learn from”, “useful”.

#### 3.1.2. Preferred Learning Style

This theme captured the perspectives on the usefulness of vicarious learning and education outcomes, and overarching view that visual learning was helpful and engaging.

P1: “Videos have been helpful”, “saw flexibility of the therapist”;

P2: “Good for learning soft skills and the application of learnt skills”;

P3: “Delivering education outcomes”;

P4: “Education was done via video demonstrations”, “as a visual learner I felt this was able to capture my attention”.

#### 3.1.3. Building Confidence

This theme captured the experience on learning-related intrapersonal factors, including confidence-building, from observation of competent peer’s skill demonstration.

P1: “Seeing CPTs (clinical psychology trainees) felt less daunting and more realistic”, “exceptionally helpful for us doing placements in the clinic”, “enhancing our learning”;

P2: “Build confidence that is required for mastery of skills in novice clinicians”.

#### 3.1.4. Putting Theory into Practice

This theme is a conglomerate of perspectives of practical learning. Participants reported on the helpfulness of observations of practical skills in clinic placement.

P1: “…Show the application of therapy approach”, “I saw flexibility of the therapist utilizing… micro-skills and… questioning styles”, “illustrate not only the skills, but gives an overview of the clinic procedures which were exceptionally helpful for us doing our placements”;

P2: “Good for learning soft skills and the application of learnt skills from modules”.

### 3.2. Quantitative Analyses

All quantitative analyses were performed using SPSS version 22.0 (IBM Corporation, New York, NY, USA). Quantitative data was analyzed using a series of six Mann-Whitney U tests to examine difference between two groups (Group: Experimental vs. Control) with scores on the NGSES-E (academic Self Efficacy) and scores on the SLO (Self-reported Learning Outcomes) as the dependent variables over three time points (Weeks 1, 3, and 6). Descriptive statistics, such as means, standard deviations, and Cronbach’s alpha values for academic SE and SLO measures are reported in Table 1.

#### Hypothesis Testing

Participants in the experimental group were hypothesized to have increased levels of academic SE and SLO as compared to participants in the control group. Six Mann-Whitney U tests were conducted to examine differences between two groups (Group: Experimental vs. control) with scores on the NGSES-E (academic Self Efficacy) and scores on the SLO (Self-reported Learning Outcomes) as the dependent variables over three time points (Week 1, 3, and 6).

In week 1, Academic SE level of the experimental group (Mean Rank = 4.00) was not significantly different to those in the control group (Mean Rank = 5.33), U = 5.00; *z* = −0.77, *p* = 0.44, two tailed. Similarly, SLO level of the experimental group (Mean Rank = 4.40) was not significantly different to those in the control group (Mean Rank = 4.67), U = 7.00; *z* = −0.15, *p* = 0.88, two tailed.

In week 3, academic SE level of the experimental group (Mean Rank = 4) was not significantly different to those in the control group (Mean Rank = 5.33), U = 5; *z* = −0.77, *p* = 0.442, two tailed. SLO level of the experimental group (Mean Rank = 5.4) was not significantly different to those in the control group (Mean Rank = 3), U = 3; *z* = −1.34, *p* = 0.180, two tailed.

In week 6, Academic SE level of the experimental group (Mean Rank = 3.9) was not significantly different to those in the control group (Mean Rank = 5.5), U = 4; *z* = −0.95, *p* = 0.341, two tailed. SLO level of the experimental group (Mean Rank = 5.3) was not significantly different to those in the control group (Mean Rank = 3.17), U = 3.5; *z* = −1.2, *p* = 0.230, two tailed.

## 4. Discussion

This study aimed to explore trainees’ perspectives on the use of a novel smartphone application in clinical psychology training through a mixed-methods study design. The qualitative study yielded four main themes: Convenience, preferred learning style, building confidence, and putting theory into practice. The theme of convenience is consistent with previous studies, which described the advantages of m-learning. Our finding was consistent with the study conducted by Al-Emran, Elsherif, and Shaalan [24]. Their study explored students’ attitudes towards m-learning and concluded that the use of m-learning in tertiary education was favourable. Similarly, our study found that m-learning offered convenience to students, with quick online access on e-platform, outside of the classroom. Such e-collaboration and flexibility in contemporary learning paradigms seems to benefit students’ needs, expectations, and learning preferences [35,36].

The theme of convenience is also consistent with Gikas and Grant’s [37] findings which reported that the ability to access information quickly was an advantage of m-learning. Similar to the current study, Gikas and Grant used a qualitative design and focus group data to explore the perspectives of tertiary students in learning with smartphones. Similar to the current study, the advantages cited were that students were able to access information quickly and conveniently on a mobile platform. In addition, Gikas and Grant reported that students had variety in learning, and situated learning. Our findings are also similar to various other studies [21,38]. Consistent with another recent qualitative study [21], the range of advantages might also include: Knowledge sharing and communication, useful and easy to learn, and personal goal setting. Such components could be fully explored in future research, and further enhancement of the application could include facilitated e-communication between the learners, as well as between the learners and the educator, and inclusion of self-monitoring of personal learning goals within the application.

The theme of preferred learning style is consistent with the findings from previous research [15,22,26]. The theme of building self-confidence is consistent with previous research [10,39]. Additionally, the theme is consistent with Heath et al.’s [40] findings that mobile devices and applications increased students’ perception of confidence with the learning content. The theme of putting theory into practice is consistent with Beauregard et al.’s [38] finding that the ease of access to practical skills on the smartphone led to increase in nurses’ SE, which enhanced their skill performance, and improved quality of care for their patients. Similarly, participants in our study commented on the helpfulness of observations of practical skills in clinical placement, which was particularly useful for clinical psychology trainees during their clinical placements. It would be good to conduct a follow-up study to explore the continued impact of their learning, as well as collect data on their perceived quality of care for patients with mental health problems (which formed the content of the video clips in the application).

## 5. Limitations

In order to strengthen the qualitative research paradigm, the researchers endeavoured to triangulate the qualitative outcomes with the quantitative findings. As such, it was anticipated that the themes of building confidence, and putting theory into practice, would be triangulated with significant increase in academic SE and SLO. However, the quantitative findings did not support the hypothesis that participants in the experimental group would have significantly increased academic SE levels and SLO as compared to participants in the control group.

The quantitative findings were not consistent with previous studies [21]. Peechapol et al. [21] found that participants’ observation of peers who performed successfully as role models led to greater academic SE, and their study supported Bandura’s theory of enhanced SE when observing competent peers and also demonstrated that smartphone application could be used as an effective learning tool for enhancing learning behaviors and academic SE. Our non-significant results might be explained by various reasons. The major limitation included a small sample size for quantitative analysis. Future study should use a larger sample size informed by a power analysis. Nevertheless, preliminary findings could be used to inform the implementation of a larger scale project involving collaborations with other universities. In our study, participants were randomly assigned to experimental and control groups. The design could be improved if both groups were matched on key characteristics. The scales used were SLO and NGSES-E, based on self-reports, and thus, subjected to recall bias and social desirability bias. Future research should supplement with objective findings, such as student grades or pass rates. Another limitation was an insufficient exploration of potential barriers of m-learning. Barriers could hinder students’ engagement with m-learning [37], and such responses could be explored using focus groups or semi-structured interviews.

In addition, there could be other extraneous factors. Students with little experience tended to be more reliant on vicarious experience to learn required skills [15], but there could be varying levels of professional experience within the pool of participants, and some participants in the control group might not be novices. However, this data was not collected in the current study. Future research could include this variable. Another extraneous variable could be gender. Recent studies that explored the role of gender differences in academic SE yielded mostly inconsistent results [12,41,42]. It is noteworthy that a local study reported that gender contributed to student’s smartphone usage and academic performance [43]. The current sample seemed to be skewed towards females. Future studies could replicate the study with a more balanced gender ratio, or incorporate gender in the analysis.

## 6. Conclusions

The qualitative study provided preliminary support from trainees’ perspectives, on some benefits of the novel smartphone application in postgraduate clinical psychology training in Singapore. The themes of convenience, preferred learning style, building confidence, and putting theory into practice are consistent with previous research that investigated students’ use of smartphone applications [27,37]. The findings are also consistent with theoretical models, such as the Cognitive theory of SE [13], vicarious learning [14], Cognitive theory of multimedia learning [17], as well as research investigating learning style preferences [18,22,44], and intrapersonal experiences of vicarious learning [15].

The overall findings should be interpreted with caution as the quantitative results were not significant. Nevertheless, the qualitative results were helpful in understanding trainees’ perspectives and experience of the novel application, indicating that future research in this innovative area is necessary. When interpreted in the context of previous research, the preliminary qualitative findings shed some light on future innovations, to inform and enhance teaching and training efforts in postgraduate clinical psychology in Singapore using mobile technology (m-learning). Clinical psychology educators in Singapore could use the preliminary qualitative findings from this study to enhance their understanding of trainees’ perspectives, for consideration when designing their teaching methodologies to cater to a range of learning styles. In view of the advent of mobile technology in our contemporary society [1,2,3,4], the inclusion of m-learning has the potential to tap upon the advantages of the mobile platform [5]. Our preliminary findings serve to encourage educators to collaborate with researchers to further innovative research in the area, and continue to gather evidence to support a contemporary step beyond the traditional classroom, in our innovation to enhance flexible and continuous learning that is not confined to the physical traditional classroom space [6].

## Figures and Tables

**Table 1:** Means, Standard Deviations, and Cronbach’s Alpha (α) of NGSES-E and SLO.

Measure	Time Points								
	Week 1 *M*	*SD*	α	Week 3 *M*	*SD*	α	Week 6 *M*	*SD*	α
SE Experimental	23.80	2.86	-	24.40	1.67	-	25.00	2.83	-
Control	26.33	3.22	-	26.00	2.65	-	26.67	2.52	-
SLO Experimental	162.00	44.39	0.90	202.00	43.68	0.91	222.00	30.95	0.92
Control	128.33	86.94	0.90	151.67	48.05	0.91	188.33	25.54	0.92

*Note.* SE = academic self-efficacy. NGSES-E = New General Self-Efficacy Scale for Education. SLO = Self-Reported Learning Outcome. Some Cronbach’s alphas cannot be calculated as all participants in that time point had provided the same score on some of the items.
